# Supplementary material for: Integrative Analysis of Expression Profiles of mRNA and MicroRNA Provides Insights of Cotton Response to Verticillium dahliae
Source: Int J Mol Sci. 2022 Apr 24;23(9):4702. doi: 10.3390/ijms23094702 (PMC9099760; doi:10.3390/ijms23094702)
Supplement: Supplementary file 1 [file ijms-23-04702-s001.zip › Supplementary Figures.pdf]

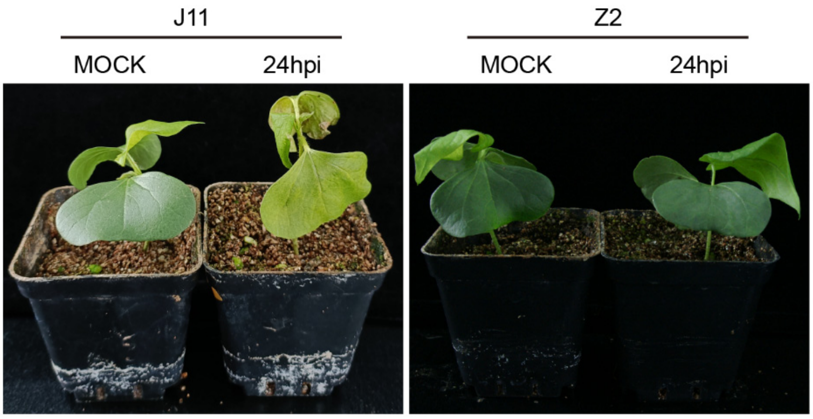

Figure S1. Phenotypes of J11 and Z2 after 20 days inoculation with *V.dahliae.*

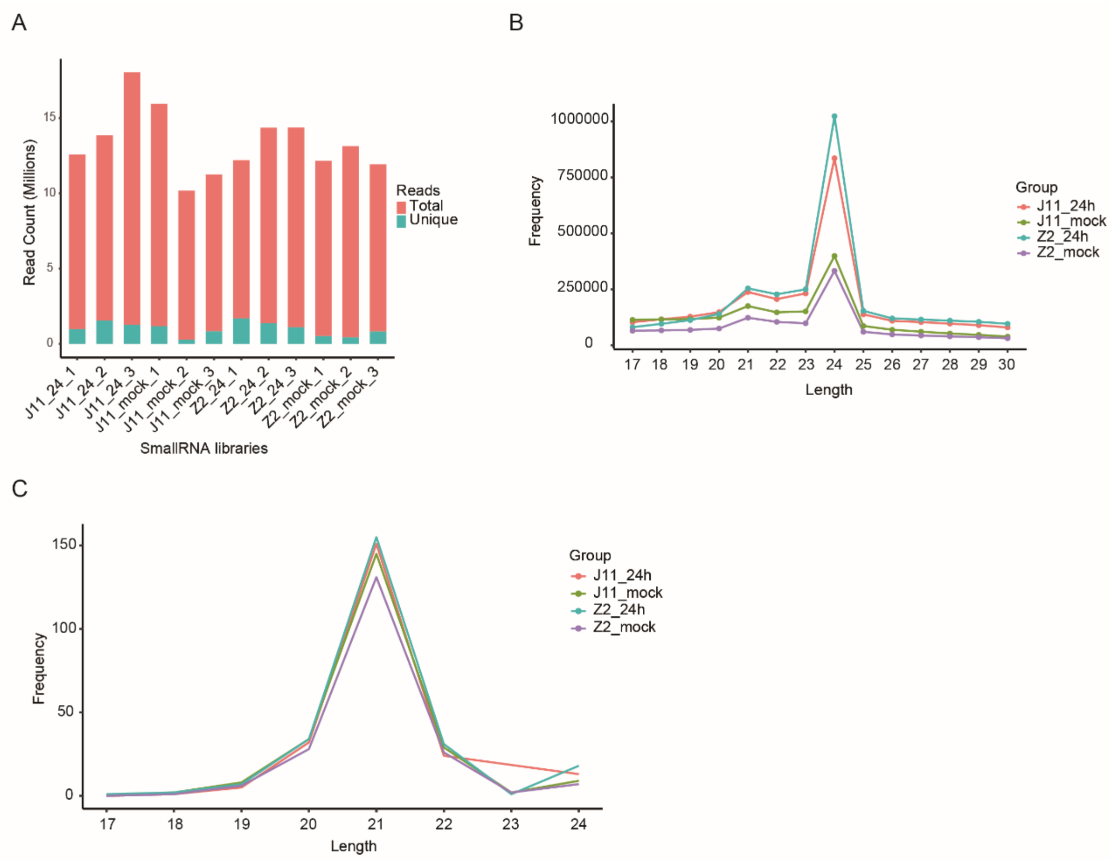

Figure S2. Cotton small RNA reads distributions. (**A**). Pre-processed reads distribution of small RNA libraries. (**B**). Length distributions of unique small RNAs in J11 and Z2. (**C**). The frequency distribution of miRNAs in J11 and Z2.

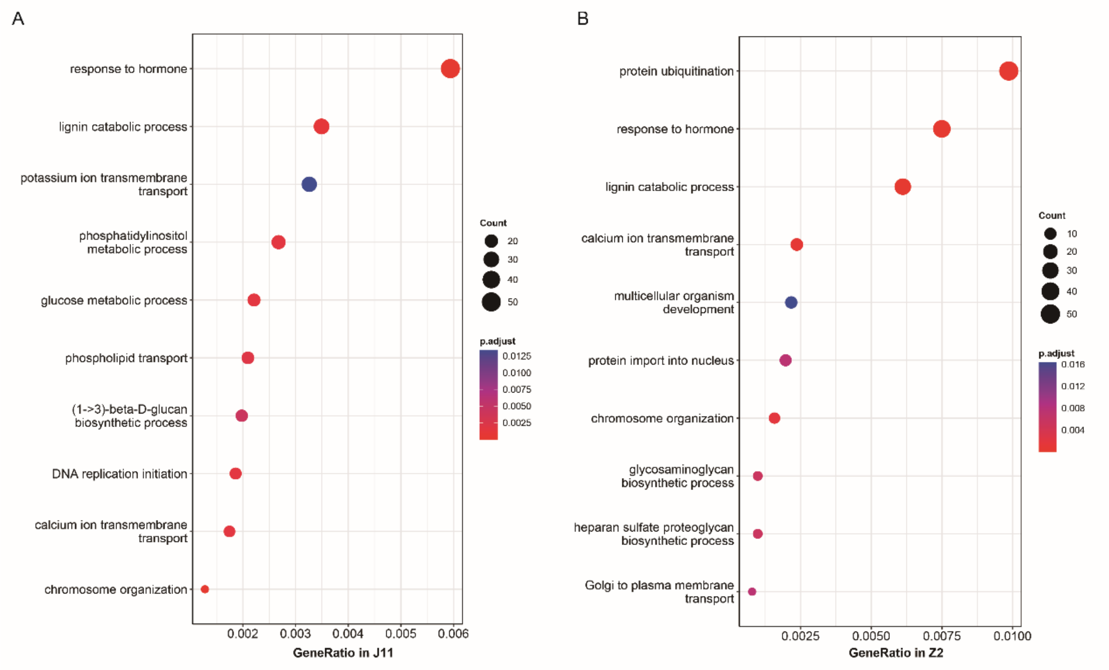

Figure S3. GO enrichment analysis of target unigenes of DEMs in J11 (**A**) and Z2 (**B**).

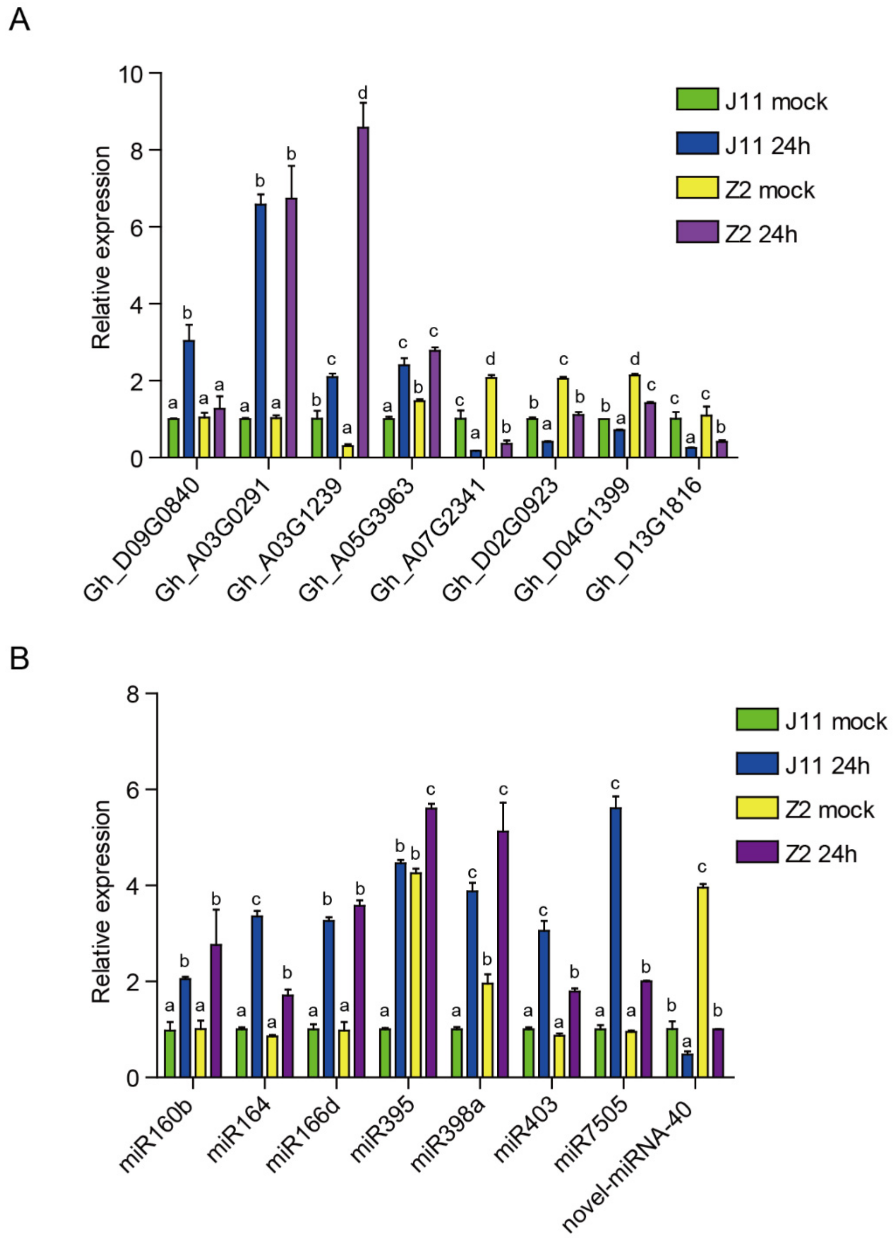

Figure S4. Quantitative real-time PCR (qRT-PCR) validation of selected unigenes and miRNAs. (**A**) qRT-PCR was performed to determine the expression levels of 8 unigenes relative to GhUBQ7. (**B**) The expression of 7 known miRNAs and 1 novel miRNA was verified by stem-loop qRT-PCR with U6 snRNA as an internal reference gene and data are means ± SD from three biological replicates. Different letters indicate significant differences, $p < 0.05$ (ANOVA test).
