# Supplementary material for: Integrative Analysis of Expression Profiles of mRNA and MicroRNA Provides Insights of Cotton Response to Verticillium dahliae
Source: Int J Mol Sci. 2022 Apr 24;23(9):4702. doi: 10.3390/ijms23094702 (PMC9099760; doi:10.3390/ijms23094702)
Supplement: Supplementary file 1 [file ijms-23-04702-s001.zip › Table S1.Summary of transcriptome sequencing results after filtering.pdf]

Table S1 Summary of transcriptome sequencing results after filtering

Sample	Clean reads	Mapping rate(%)	Effective rate(%)	Q30(%)
J11 mock-1	18,919,087	89.34	93.16	92.43
J11 mock-2	18,613,300	89.37	93.28	93.62
J11 mock-3	19,327,874	88.90	95.09	92.41
J11 24h-1	20,125,921	74.01	93.43	92.56
J11 24h-2	20,901,469	79.65	94.74	92.59
J11 24h-3	21,810,013	73.62	94.30	92.11
Z2 mock-1	20,137,350	94.79	93.74	92.27
Z2 mock-2	22,658,917	93.43	92.15	92.28
Z2 mock-3	21,816,358	95.20	94.11	92.38
Z2 24h-1	24,645,435	77.93	95.16	92.64
Z2 24h-2	24,087,659	76.05	94.95	92.37
Z2 24h-3	21,823,327	79.84	95.03	92.41
